# A Deceptively Unremarkable Standstill: A Case Report of a Rare Cardiac Electrophysiologic Event

**DOI:** 10.7759/cureus.33763

**Published:** 2023-01-14

**Authors:** Kofi Seffah, Walter Y Agyeman, Jaime Cardona, Patrick Berchie

**Affiliations:** 1 Internal Medicine, Piedmont Athens Regional, Athens, USA

**Keywords:** chest pain, recurrent, syncope, telemetry, ventricular standstill, standstill

## Abstract

Ventricular standstill is a rare cardiac event associated with a high mortality. It is considered a ventricular fibrillation equivalent. The longer the duration, the poorer the prognosis. It is therefore unusual for an individual to have recurrent episodes of standstill and survive, without morbidity and rapid mortality. Here, we report the unique case of a 67-year-old male, previously diagnosed with heart disease, requiring intervention, who lived with recurrent syncopal episodes for a decade. Though such occurrences have previously been documented, we seek to stress the importance of using clinical tools in assessing what could easily have been passed off as orthostatic in origin.

## Introduction

Ventricular standstill is a potentially lethal electrophysiologic phenomenon appearing on the EKG as P waves in the absence of QRS complexes. Slow ventricular escape rhythms with broad QRS complexes may also be observed. It usually results in syncope from cardiac arrest with a high risk of mortality [1,2].

We present a patient with recurrent episodes of pre-syncope, ongoing for almost a decade, who was eventually diagnosed with a ventricular standstill. This is an unusual presentation of long-term survival and is unique in the medical literature. In this case presentation, we seek to find out if there were any patient characteristics that promoted this survival. Also, we seek to encourage the standardization and use of clinical risk assessment tools in evaluating undecided syncopal episodes, as this may be lifesaving.

## Case presentation

A 67-year-old male with a past medical history of hyperlipidemia and unspecified heart disease presented to the hospital in November 2022 following a spell of pre-syncope, described as dizziness, nausea, and a sense of impending loss of consciousness. Records indicate that in 2013 he had been evaluated by a cardiologist following similar complaints. A stress test carried out t that time returned abnormal (the stress test report could not be obtained). He was informed that he would need an invasive procedure for further investigation and treatment, but the patient declined, opting for lifestyle changes instead. He was able to lose 30 pounds of weight over a two-year period, on a plant-based diet. He, however, continued to experience intermittent weakness and brief spells of fatigue. The patient followed up with his primary physician following these episodes. But neither bradycardia nor an arrhythmia was noted at any time in visits. There was no documented concern for an urgent review by a cardiologist for his symptoms.

About four months prior to his presentation, the patient was reported to have been rescued from a pool, found semi-conscious. He required no chest compressions nor advanced life support. He only felt weak and deferred his presentation to the hospital. He denied unilateral weakness, palpitations, or chest pain associated with his bouts of dizziness. He denied vertigo, aphasia, nausea, or vomiting. He would subsequently have several more episodes: whilst seated, with minimal activity (reaching for a wallet on the ground, watching television), and some episodes waking him up from sleep with a sense of impending doom.

**Figure 1:**
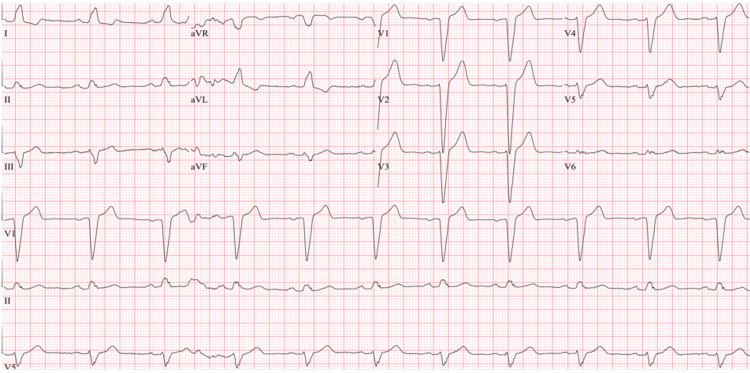
EKG on admission: ventricular rate 60 bpm, sinus rhythm, left axis deviation, prolonged PR intervals in all leads, with left bundle branch block

In his penultimate emergency room visit, his symptoms were deemed to be orthostatic, and the patient was discharged with reassurance and a follow-up appointment with a cardiologist. He returned only two days later with a recurrence of said light-headedness, unusually more intense than usual. His EKG showed a left bundle branch block (LBBB) (Figure 1) and the patient was placed under observation with telemetry. The documenting physician was not convinced this was a new LBBB. 

**Figure 2:**
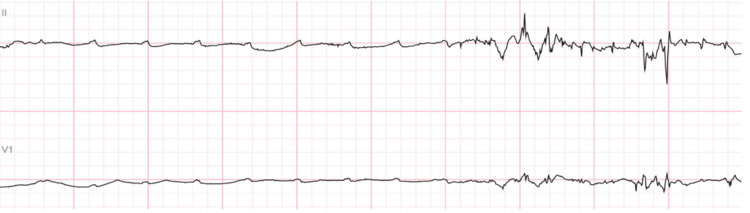
Telemetry strip showing 10 seconds that include presence of P waves with no QRS complexes present for about seven seconds. Captured during the 17-second event.

Our patient continued to experience episodes of pre-syncope during the day, where, after waking from sleep, he suddenly felt "all his blood draining," as he stretched from bed. A telemetry pause of 17 seconds was noted (Figure 2), commensurate with his symptoms, and a cardiology consult was placed. The patient was reviewed and noted to meet the criteria for permanent pacemaker insertion. He received the device the same day and was discharged home the next.

## Discussion

Paroxysmal ventricular standstill is a rare cardiac electrophysiologic event with danger levels akin or even higher than ventricular fibrillation [2]. Although there have been previous reports in the literature, it was first described and named by Harold Cookson in 1952 as a heart rhythm causing a Stokes-Adams attack [3]. It was also described by Hick in 1954 in a patient who presented with syncope and convulsions and was shown to have spontaneous recurrent attacks of ventricular standstill without preceding or subsequent partial heart block [4]. They usually present as syncope and even sudden cardiac death, with standstills of more than 10 seconds considered to be 10 times more dangerous than ventricular fibrillation. They appear as P waves without QRS complexes as was seen in the patient presented in this report. Generally, there is no loss of sinoatrial integrity [1]; however, no supraventricular impulses are transmitted to the ventricles and ventricular automaticity foci appear to be blunted with the absence of an escape rhythm. This functional depression of idioventricular automaticity results in a loss of cardiac output [4]. The causes of paroxysmal ventricular standstill include electrolyte imbalances, acidosis, cardiogenic shock, degeneration of the conduction pathways, increased vagal tone, and ischemic heart disease. Medications such as amiodarone, beta-blockers, calcium channel blockers, and digoxin can also precipitate a ventricular standstill [1].

The patient presented here had likely experienced these conduction defects for almost a decade, as he had been notified by a cardiologist following his positive stress test of the need for an invasive procedure. An alternate explanation could likely be a progressive ischemic disease, which reached its nadir at the time he presented to us. This assertion is supported by the EKG findings of the LBBB. What makes this case unique is the duration of his symptoms for a decade and how long the patient survived, despite the acknowledged poor prognosis associated with sudden cardiac death [5].

American Heart Association (AHA)/American College of Cardiology (ACC) guidelines of 1998 stated that sustained pause-dependent VT was a class I indication for pacemaker placement [6]. However, there is no mention of this indication in the revised edition of 2018 [7], although this latter guideline states a pacemaker may be placed in the event of a variety of reasons, including symptomatic bradycardia. Prior to his last admission, the patient did not meet the criteria for pacemaker placement, and the only suggestive finding was the stress test, which was not followed up on with further investigation as recommended. 

Recommendations have never sought to replace clinical judgment but rather lend expediency to clinical decision-making [7]. The patient presented here always had normal sinus rhythms during visits to his PCP, with no evidence of bradycardia, obviating the need for pacing. His history of "heart disease" alone did not warrant intervention on the face of it. There were, however, a few red flags that constitute enough reason to at least require an event monitor. His history of recurrent bouts of loss of consciousness even whilst seated, a previous diagnosis of "cardiac disease," and symptoms unrelated to exertion ideally should have triggered further work-up. Screening tools such as the San Francisco screening tool [8], the OESIL (Osservatorio Epidemiologico sulla Sincope nel Lazio) screening tool [9], or the ROSE (Risk Stratification of Syncope in the Emergency Department) screen [10] would clearly have lent expediency to a cardiogenic etiology and warranted further work-up. In particular, the San Francisco score places this patient at high risk of cardiogenic syncope, implying the need for an intervention.

Features of cardiogenic syncope that should have lent more urgency prior to the final discovery of the ventricular standstill include previously untriggered dizziness, prior heart disease, and the repeated nature [5]. The absence of a prior diagnosis of a seizure disorder may have supported this as well [11]. 

## Conclusions

Whilst healthy lifestyle recommendations, such as weight loss and high vegetable diets may promote overall longevity, there is no clear indication that there is a reversal of or slowing down of fatal arrhythmias. Clinical tools should be employed routinely to assist in the evaluation of syncope and factored in clinical decision-making regardless of seeming simplicity. A normal initial EKG in a patient with recurrent syncopal events does not exclude a cardiac cause. As a clinical entity, ventricular standstill may present with features suggestive of another etiology, including seizure disorder, vasovagal, or orthostatic, and as a master of disguise. A high index of suspicion and clinical judgment is required for the diagnosis of this unique cardiac phenomenon.
